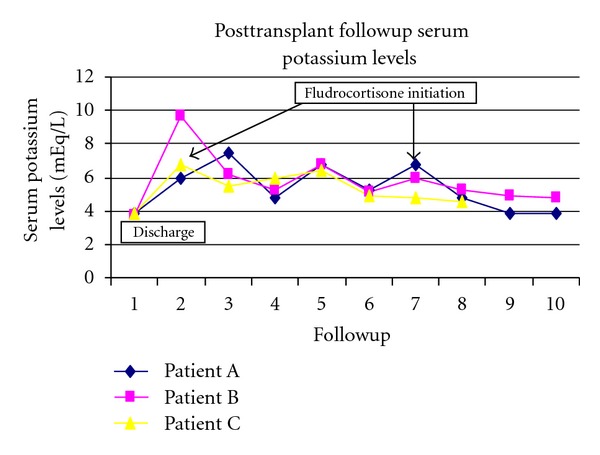# Fludrocortisone Therapy in Renal Transplant Recipients with Persistent Hyperkalemia

**DOI:** 10.1155/2012/586859

**Published:** 2012-09-16

**Authors:** K. Marfo, D. Glicklich

**Affiliations:** ^1^Department of Pharmacy & Abdominal Organ Transplantation, Montefiore Medical Center, The University Hospital of Albert Einstein College of Medicine, 111 East 210th Street, Bronx, NY 10467, USA; ^2^Department of Medicine and Transplant Nephrology, Montefiore Medical Center, Bronx, NY 10467, USA

## Abstract

Hyperkalemia after kidney transplantation is a common electrolyte disturbance and the risk factors are multifactorial. Pharmacotherapeutic agents for chronic management of hyperkalemia in kidney transplant patients may be relatively contraindicated or provide suboptimal efficacy. Fludrocortisone, an endogenous mineralocorticoid mimics the actions of aldosterone, hence hyperkalemia reversal. We describe three- case series of persistent hyperkalemia with demonstrated benefit from fludrocortisone therapy. Our three renal transplant recipients with multiple emergency room visits for elevated serum potassium levels despite treatment with diuretics, sodium bicarbonate, and sodium polystyrene sulfonate responded well to fludrocortisones therapy. Upon fludrocortisone initiation and maintenance therapy, all three patients experienced a decline in serum potassium levels to normal reference range.

## 1. Introduction

Hyperkalemia is commonly observed in renal transplant recipients, with an incidence of 44%–73% in patients maintained on calcineurin inhibitors [1]. These drugs are associated with a syndrome similar to hyporenin hypoaldosteronism with decreased aldosterone release, impairment of tubular potassium secretion via inhibition of sodium/potassium ATPase activity in the medullary thick ascending limb and collecting ducts, and decreased expression of the mineralocorticoid receptor [2–6]. Renal insufficiency and other drugs such as trimethoprim, angiotensin-converting enzyme inhibitors, angiotensin receptor blockers, and beta-adrenergic blockers may also contribute to the hyperkalemia seen in renal transplant recipients. In this setting hyperkalemia is commonly treated by reduction or discontinuation of the suspected medication, dietary modification, and addition of thiazide or loop diuretics, sodium bicarbonate, and sodium polystyrene sulfonate (SPS). Mineralocorticoids (i.e., fludrocortisones) facilitate sodium resorption and promote potassium excretion at the level of the distal renal tubule, hence its potential for use in management of hyperkalemia. We were able to find only one case report of fludrocortisone used to successfully manage severe hyperkalemia refractory to other measures in renal transplant recipients [7]. Therefore we are reporting our recent experience with fludrocortisone to treat hyperkalemia in three renal transplant patients.

## 2. Case Reports

Patient A is a 66-year-old woman with end-stage renal disease secondary to diabetes mellitus and hypertension who underwent cadaveric kidney transplantation. Upon discharge the patient's serum creatinine was 4.5 mg/dL, serum potassium was 3.8 mEq/L, and serum bicarbonate was 20 mEq/L. She was discharged home with maintenance immunosuppressive therapy with mycophenolate mofetil, tacrolimus, and prednisone and, furosemide (80 mg/day), sulfamethoxazole-trimethoprim (SMZ/TMP 400–80 mg/day), and sodium bicarbonate (1300 mg/day). 

On post-op day 20, patient A's serum potassium was 5.9 mEq/L. At this time, she was started on metolazone (5 mg/day) and continued on furosemide, SMZ/TMP, and sodium bicarbonate. During a clinic visit on post-op day 53, patient A was taken to the emergency room with complaints of tremor, weakness, and palpitations. In clinic, she was found to have an elevated potassium level of 7.5 mEq/L. An EKG was performed and no changes were found. In the emergency room, she was treated with 30 grams of SPS, and her potassium level decreased to 4.8 mEq/L. 

Following her visit to the ER, patient A was started on SPS to control her hyperkalemia (5 grams/week) for 3 weeks. She also continued taking furosemide (80 mg/day), metolazone (5 mg/day), SMZ/TMP (400–80 mg/day), and sodium bicarbonate (3.9 g/day). On post-op day 75, the patient was again found to have an elevated serum potassium level of 6.7 mEq/L. Patient A was instructed to take 45 grams of SPS and to return to the clinic the following day. Her potassium level decreased to 5.2 mEq/L and the patient was started on hydrochlorothiazide 25 mg daily. Her dose of SPS was increased to 15 grams per week and furosemide was cut in half (40 mg/day). 

Several weeks later on post-op day 117, patient A was again admitted to the hospital from clinic for elevated potassium level of 6.8 mEq/L. Her serum creatinine was 2.3 mg/dL and serum bicarbonate was 20 mEq/L. She was treated with SPS (30 g), IV fluids, sodium bicarbonate (50 mEq), dextrose (25 g), and insulin (10 units), which brought her serum potassium level to 4.8 mEq/L. On post-op day 119, she was started on fludrocortisone 0.1 mg daily and her dose of hydrochlorothiazide was increased (50 mg/day). Metolazone and sulfamethoxazole-trimethoprim were discontinued and furosemide was decreased (20 mg daily).

Fludrocortisone was continued for five months. During this time, her serum potassium levels were within reference range of 3.5–5.5 mEq/L. 

Patient B is a 64-year-old man with end-stage renal disease secondary to diabetes mellitus and hypertension who also underwent a cadaveric renal transplant at our Transplant Center. Maintenance immunosuppressive therapy with tacrolimus, mycophenolate mofetil, and prednisone was started and continued upon discharge. He was also started on SMZ/TMP (400–80 mg/day) for PCP prophylaxis and furosemide (40 mg/d). Upon discharge on post-op day 7, his serum creatinine level was 4.3 mg/dL and serum potassium level was 3.7 mEq/L. 

Three weeks later on post-op day 20, the patient was sent to the ER from clinic due to an elevated potassium level of 9.7 mEq/L. The patient did not have any complaints of chest pains, muscle weakness, or shortness of breath. An EKG was done which showed no significant findings. In the ER he received 30 grams of SPS. The repeat potassium levels two and four hours later where 6.2 mEq/L and 5.2 mEq/L, respectively. When the patient was discharged, furosemide was discontinued and hydrochlorothiazide (25 mg/day), sodium bicarbonate (3.9 g/day), and SPS (15 g as needed) were started. 

On post-op day 62, the patient was sent to the emergency room again from clinic for an elevated serum potassium level of 6.7 mEq/L. In the ER, he was treated with 30 grams of SPS. He was discharged from the ED with a potassium level of 5.1 mEq/L. 

After returning to the clinic on post-op day 65, he was started on fludrocortisone for treatment of hyperkalemia (0.1 mg/day). At the time fludrocortisone was initiated, serum potassium level was 5.9 mEq/L, serum creatinine was 2.5 mg/dL, and serum bicarbonate was 28 mEq/L. Patient B has been on fludrocortisone for 16 weeks. During that time, he did not have an elevated serum potassium level greater than 5.5 mEq/L. His last serum potassium on post-op day 174 was 5.2 mEq/L.

Patient C is a 42-year-old man with end-stage renal disease secondary to polycystic kidney disease who underwent a cadaveric renal transplant and a simultaneous bilateral nephrectomy. His past medical history is significant for hypertension. The patient was started on maintenance immunosuppression with tacrolimus, mycophenolate sodium, and prednisone. The patient was also given SMZ/TMP (400–80 mg/day) and furosemide (160 mg/day). Patient C was discharged on post-op day 13 with a serum creatinine level of 7.8 mg/dL and the serum potassium level was 3.8 mEq/L.

Furosemide was also decreased (80 mg/day). During a clinic visit on post-op day 31, patient C was found to have an elevated serum potassium level of 6.8 mEq/L. He was sent to the ER and admitted to the transplant service. An EKG was performed which showed some T wave abnormalities. He was treated with SPS (60 grams), sodium bicarbonate (50 mEq), calcium gluconate (250 mg IV), dextrose (25 grams), and insulin (10 units). During this admission, he was started on fludrocortisone 0.1 mg daily and sodium bicarbonate (3.9 grams/day). He was continued on SMZ/TMP (400–80 mg/day) and furosemide (80 mg/day). At discharge on post-op day 32, his serum potassium level was 5.5 mEq/L and serum bicarbonate was 19 mEq/L. 

Patient C continued taking fludrocortisone to control his hyperkalemia for three weeks. On post-op day 54, the patient's serum potassium level rose to 5.9 mEq/L. Until this time the patient's hyperkalemia had been controlled with no serum potassium levels greater than 5.5 mEq/L. Patient C told the team he had not been complianed with fludrocortisone and had missed three days of treatment. After a new prescription was written, he resumed treatment. However two weeks later on post-op day 68, the patient was found to have an elevated serum potassium level of 6.4 mEq/L. After treatment with 15 grams of SPS, the patient's serum potassium level decreased to 5.6 mEq/L. The patient was started on hydrochlorothiazide (25 mg/day) and continued on the fludrocortisone (0.1 mg/day). His last serum potassium level on post-op day 81, while maintained on this regimen, was 4.9 mEq/L.

## 3. Discussion

Our three patients described in this report all had severe hyperkalemia requiring multiple readmissions to the hospital despite ongoing therapy with diuretics, sodium bicarbonate, and SPS. In each patient addition of low dose fludrocortisone was successful in controlling the serum potassium, allowed discontinuation of sodium polystyrene, and prevented further need for emergency room or hospital admission for hyperkalemia (Figure 1). Our patients developed hyperkalemia within the first 3 months after transplant when calcineurin levels have to be higher, SMZ/TMP prophylaxis is common, and the transplant kidney may still be recovering from reperfusion injury. 

There is only one previous report of fludrocortisone therapy in a renal transplant recipient. Rangel et al. [7] reported a patient who developed severe hyperkalemia due to type 4 renal tubular acidosis with hypoaldosteronism 3 months after transplant. This patient had a stable serum creatinine of 1.7 mg/dL for more than 4 years on 0.1 mg per day of fludrocortisone. During this time, blood pressure was well controlled. Unfortunately, there was no mention of urine protein measurements. Over the four-year period, he was also maintained on hydrochlorothiazide, sodium bicarbonate, prednisone, tacrolimus, mycophenolate mofetil, propranolol, and enalapril.

In experimental models of chronic renal failure, aldosterone has been implicated in worsening hypertension, proteinuria, interstitial inflammation, and fibrosis. In human diabetics with albuminuria and proteinuria aldosterone blockers reduce the proteinuria, and albuminuria [8–11]. As fludorocortisone is an analogue of aldosterone, the drug theoretically may have long-term undesirable effects on the renal allograft but there are no such published data. 

Fludrocortisone has potent mineralocorticoid properties and as a result, most adverse reactions are due to its mineralocorticoid activity. These adverse effects include fluid imbalance, edema, congestive heart failure, and hypertension. Glucocorticoid side effects of fludrocortisone such as muscle weakness, steroid myopathy, loss of muscle mass, and osteoporosis may also occur when the drug is used over a prolonged period of time or in conjunction with a glucocorticoid. In our current case series, fludrocortisone was not associated with exacerbation of hypertension, proteinuria, or worsening renal function. Thus, in our experience low dose fludrocortisone used in the early posttransplant period in association with diuretics and sodium bicarbonate, was safe and effective in preventing severe hyperkalemia. However, in patients with underlying congestive heart failure, fludrocortisone therapy should be used with caution due to its negative impact on hemodynamic parameters. Prospective randomized trials are necessary to determine the long-term efficacy and safety of fludrocortisone therapy for management of hyperkalemia in renal transplant recipients.

## Figures and Tables

**Figure 1:**